# Medical Society of the County of Albany

**Published:** 1871-05

**Authors:** James S. Bailey


BUFFALO

### Medical and Surgical Journal.

VOL. X. MAY, 1871. No. 10.

### Original Communications.

ART. I.—*Medical Society of the County of Albany.* Semi-monthly Meeting, March 28th, 1871.

REPORTED BY JAMES S. BAILEY, M. D.

Dr. WM. H. CRAIG in the Chair.

Dr. J. W. MOORE presented a specimen of flexor sublimis muscle, 8 inches in length, which was torn from the arm, by the wrist of a woman being caught in a hook and forcibly detached.

Dr. E. R. HUN moved that Dr. Lambert, of Salem, be requested to read his paper.

Dr. LAMBERT then read a report of a case of disease of both kidneys. He first saw the patient in Dec. 1862, aged 33, married, nervous temperament, weight 115 lbs., habits good; was suffering from a painful swelling in the right hypochondrium, and in the region of the right kidney; and had also suffered for eight years from painful and difficult micturition, with occasional hæmaturia and abundant brick red deposits; general health not greatly impaired. Diagnosis: Abcess of kidney. The tumor enlarged, and was opened January 11th; three pints of purulent matter escaped. He resumed his business in eight weeks, the opening healing in two months. From this time his health was apparently as good as usual—virility impaired. In March, 1870, experienced weight, pain and tenderness in left loin, with an increase in urinary difficulties; general health failing. April 20th, slight tumefaction and considerable tenderness over kidneys, on pressure. July 25th, symptoms more decided with excruciating pain in vicinity of kidneys, extending to the bladder and extremity of urethra; a sense, and weight, and dragging in the perineum; urine scanty and voided frequently; appetite almost totally lost; nausea and vomiting became permanent symptoms, and continued to the end; but little relief obtained from anodynes and hypnotics. The tumor on the left hypochondrium well defined: it was covered with a perceptible bulging of the loin posteriorly. Diagnosis: Suppurative disease of the kidney. Prognosis unfavorable. Early in October a white, creamy deposit was observed in the urine, which gradually increased to two-sixths ounces per day; urine slightly albuminous; cystiles appeared evidently developed by the presence and decomposition of pus and urine. Would not allow a catheter or sound to be introduced during his illness. Directly on the free passage of pus, per urethra, the tumor in the hypochondrium abated, as did the severe painful symptoms. Amount of urine voided, 16 to 28 f℥ per day, —sp. gr. 1.012 to 1.020—until about ten days of death, when it was reduced to 6 to 10 f℥, and was passed with great suffering. Indications of uræmic poisoning not manifested until the last three or four weeks of life, and then only in a mild form. Conscious, and able to urinate, till a few minutes before death. Died without a struggle, Dec. 24th.

*Sectio Cadaveris* twelve hours after death: Body greatly emaciated; rigor mortis moderate; walls of abdomen thin, and contained only a trace of adipose tissue. The vicera appeared in their natural position, except in the left hypochondrium, where they were displaced by a large tumor, which bulged high up in the abdominal cavity, over which the descending colon was found lying very much contracted, and adhered for about five inches in extent. The tumor was the left kidney greatly enlarged and firmly adherent. The fibrous adhesions were so firm that the entire organ required to be dissected out before removal. It had an elastic boggy feel, and measured 5¾ inches in length, 4 inches in breadth, and 3¾ inches in thickness, and weighed 22 ounces. Its capsule was much thickened, (it was 1¼ inch thick), and adherent to the surrounding tissues, easily detached from the kidney, leaving a smooth surface. The greater portion of the kidney was degenerated into a white, cheesy substance, and contained several large abcesses filled with pus, opening into the pelvis. A portion of renal substance, equal to about one-third of a normal kidney, appeared rather pale. The pelvis was almost obliterated, and its contracted cavity filled with cheesy substance, mingled with pus and urine. Urethral coats thickened, and its cavity dilated ; at the termination of the urethra in the bladder, its membranes was found ulcerated, and presented a a granular appearance.

The right kidney was firmly adherent posteriorly in apposition with the cicatrix of an external opening made in January, 1863. The kidney was degenerated into two fibrous bodies loosely connected, and measuring 1½ inches in diameter, together weighing 2½ ounces ; capsule firmly adherent to kidney. The cut surface of the two bodies presented a firm, compact and shining tissue ; ureter was occluded ; the bladder contained about six ounces of urine and pus ; its coats were greatly thickened, and the lining membrane extensively ulcerated. No calculus was found in the bladder or kidneys. Liver or spleen slightly enlarged, but apparently healthy· The other abdominal organs were in a noımal condition. No farther examination was permitted. Dr. Stephen Rogers examined the kidneys, and found that the portion of the larger kidney shows that it had been invaded by both instertitial and tubercular inflammation. It appears to have been the seat of a most destructive pyelo-nephritis. The other kidney presented the condition known as renal cirrhosis.

Dr. C. H. PORTER then offered the following:

*Resolved,* That the thanks of this Society are hereby offered to Dr. John Lambert for his promptness in reading, before this Society, his report of a case of Disease of both Kidneys ; and that a copy of the same be requested for the Society. Carried.

Dr. E. R. HUN stated that he had examined the kidneys microscopically. The larger one was an example of pure fatty degeneration : the smaller one was cirrhorsed. The larger one contained traces of what was apparently tuberculous matter, certainly of some foreign substance. Last summer he stated he had two similar cases, both containing a large quantity of a creamy substance. In one patient there was tubercular disease of both lungs. The patient from whom the second specimen was taken had had tuberculosis of the lungs fifteen years before, and had apparently recovered; cicatrices were formed in both lungs. Dr. Alonzo Clark considered that the condition in which the kidneys were found was the result of pyelitis; others, however, did not hold this view. He believed that the pathological condition of the kidneys in Dr. Lambert's case was the result of tuberculosis.

Dr. C. A. ROBERTSON then reported the following cases of Degenerative Diseases of the Kidneys detected by Opthalmoscopic observation of the Fundus Oculi. Mrs. M. M., age 71, small, thin, complained only of "salt rheum," or psoriasis, of long standing. Remarked that her strength was somewhat diminished, and said she was compelled to void her urine several times at night, and had some uneasiness in the back below the region of the kidneys. Vision seriously impaired; opthalmoscopic examination showed retina choroidal exudation; urine contained waxy tubular casts, epthelial scales, and hyphosphatic crystals in abundance. Diagnosis: Parenchymatous degeneration of the kidney. Prognosis grave; no disease of the kidneys had been suspected before.

Mr. H. M., aet. 29, a heavy, lumpy, amorphous German. Drove a team in the old country, walking the entire day. Drank wine and lager freely; feet swelled occasionally after work. Last summer found beer to affect him, causing unpleasant symptons in the head. Refrained from stimulants, only taking on Sunday two glasses of lager. Thirst intense; anasarca of feet and legs; felt weak; had now a pasty appearance. Within six weeks sight growing dim; now useless in one eye; could only count three feet off with better eye. Opthalmoscope showed fundus of the eye largely invaded, with exudatie patches in choroid and retina, and effusions of blood in several spots. Urine contained albumen, abundant oil globules, and epthelial scales. Diagnosis: Amyloid degeneration of kidneys. Prognosis more favorable than in the former case. Treatment: Iron and bark, and an absolute milk diet.

Dr. J. L. BABCOCK said that Dr. Lewi was detained, and had requested him to present a statement of the case which he (Dr. Lewi) was to have reported; he would therefore give a brief epitome of it. The patient had excessive flooding, which it was supposed arose from a premature labor. On digital examination, a soft, spongy body was found lying partly external to the os uteri. Two fingers were inserted in the os, and passed completely around its circumference. The fingers came in contact with what the Doctor believed to be the funis of a placenta. The hemorrhage, after a time, ceased; and, since that time, now six weeks, there has been no labor pains or expulsion of the foetus, and the patient was apparently well. Dr. Babcock stated he had known the placenta retained for six weeks after delivery, but had never heard of such a case as that of Dr. Lewi's.

Other members regarded it as possible that there might be a mistake in the diagnosis, but thought that the facts presented would not justify a positive opinion.

The Society then adjourned.

---

MEDICAL SOCIETY OF THE COUNTY OF ALBANY,
SPECIAL MEETING, April 6th, 1871.

Dr. W. H. BAILEY, President, in the Chair, said: *Gentlemen,*—It becomes my painful duty to announce the sad intelligence of the death of Dr. ALEXANDER EDMESTON. He died yesterday, at half-past two, at his residence, after a lingering illness of several years. We have met to pay proper respect to his memory, and to express our sorrow that another vacancy has been made in our ranks. During the early part of the late rebellion, stimulated by a desire to render his country the service due from every patriot, Dr. Edmerston was one of the first medical officers that joined the army. This is not the time to dwell upon the faithfulness with which he discharged every duty through that fearful struggle, nor of the appreciation of such service by the government in his promotion, and in placing him in responsible position. It was in this fraternal contest that he contracted the disease that finally proved fatal. Gentlemen, take such action as you think suitable on this melancholy occasion.

Dr. Wm. H. CRAIG said: *Mr. President,*—I should feel myself derilect to duty, as well as fail to give expression to my own feelings, were I to allow this occasion to pass without making some expression of the sorrow I feel, in common with you all, at this time. Dr. Alexander Edmeston died from an attack of hemorrhage from the bowels. His illness followed him ever since he left the army, where he contracted his disease, which frequently confined him to his house for several weeks at a time. The last attack lasted longer than former ones, although not characterized by as much acute pain. He gave promise of recovery until within a few hours of his death. I have known Dr. Edmeston since our student days, and have had occasion to see him frequently in council in regard to his disease, in common with many other physicians, and can bear most cheerful testimony to his worth as an able physician and valuable citizen.

Dr. Edmeston was a man of great energy of character, which sometimes caused him to overtask his physical strength. No matter what he undertook, whether it was the discharge of professional duties, public enterprise, or the requirements of social life, he exhibited the same untiring energy and enthusiasm.

He was one of the first to respond to the call of his country and the last to return home; and he has now given up his life as a sacrifice to his patriotic devotion. And assembled as we are, to-day, to pay a last tribute to his memory as a valuable member of this Society, as an esteemed physician and a worthy citizen, we can at best fully express the sense of our loss, and reflect, in fitting words of condolence, the public sorrow.

Dr. Edmeston has occupied an important public position as Supervisor of his Ward, and discharged its duties with vigilence and fidelity.

We would, in common with a bereaved community, express our sympathy with his afflicted family, and bear this public testimony to his worth.

Dr. STEVENS said: *Mr. President and Gentlemen*,—It is with deep sorrow that I rise to speak of the associate and friend who has so recently passed from us for all time. We have lost a colleague who, by his courteous manner, his jealous friendship, and his remarkable energy, had endeared himself not only to his co-workers in his profession, but to a very large circle of friends. As I recall the history of my own acquaintance with Dr. Edmeston, it is with unsullied satisfaction with its continuance, and with deep grief at the severance. When, some five years since, I came to this city a stranger, I learned that Dr. Edmeston had been for a short time connected with the army corps of which I was a member, I soon became acquainted with him, and have since that time known him intimately. I have watched him in his pleasant social relations, in his kind and sympathetic care for the sick, in the excitement of political struggle, in the sufferings of more than two years of most painful illness, and in the dark hours of death, yet I have never known a departure from that urbanity of manner which so eminently characterized his association with all classes of men. His intercourse with his friends was characterized by the warmest regard, while to those who were in any way opposed to him he manifested only kindness and courtesy. It is now about two and a half years since I was summoned in haste to attend Dr. Edmeston in a sudden and violent attack of sickness. In a day or two he was sufficiently recovered to resume his business, but was by no means well. Soon another attack prostrated him, and again he resumed his business with redoubled energy. About the middle of December, 1868, he was, for the third time, brought to his bed by severe illness. This time he remained confined to his room for some ten days. From that time forward, Dr. Edmeston was a confirmed invalid. Rallying from a severe illness, he would enter upon the duties of his profession with all the zeal and energy which had characterized his day of robust health, only to be suddenly prostrated by the most violent suffering.

About a year ago Dr. Craig became associated with me in attendance upon him, and aided by the wise counsels of Drs. James McNaughton and Thomas Hun, we have together labored, if possible, to arrest the progress of his disease.

While in the army he had contracted chronic diarrhoea, from which he has never been free, until during the last year, when it seemed to alternate with obstinate constipation. His sufferings seemed to arise from an inflamed and doubtless ulcerated condition of the bowels—a condition, in all probability, resulting from the chronic disease acquired in Southern lagoons.

During the last year he had several attacks of violent hemorrhage from the bowels, at which time he seemed upon the verge of death. From his attack on Tuesday last, although extremely low, we hoped that, as at other times, the hemorrhage would cease, and that his strength might again rally, but our hopes were cut off—the vital flame became lower and dimmer until, at half-past ten yesterday, Dr. Edmeston was dead.

He has left a sorrowing wife who, throughout all his long illness, has watched over him, and ministered to his many wants, with that devotion which could only have been sustained by the most ardent affection; a mother, whose idol he had ever been; relatives, to whom he was bound by most endearing ties; friends, in great numbers, who loved him most earnestly; and a profession in which he was a most zealous and earnest worker. In our sorrow for our great loss, we have the consolation that our friend has closed his useful and active life only to go to his reward.

Dr. STEVENS then offered the following:

*Whereas*, It has pleased Him, in whose hands are the ingoings and the outgoings of life, to remove from our midst our esteemed friend and colleague, Dr. Alexander A. Edmeston.

*Resolved*, That we have heard with deep sorrow of the death of our late beloved associate, who has been cut down in the midst of a most active and useful life, by which event we are deprived of our most active and efficient member.

*Resolved*, That, while we remember his unvarying kindness and courtesy in his relations with his professional colleagues, we cannot but regard his loss as a deep personal affliction to each.

*Resolved*, That, in our heart-felt sorrow for our great loss, we extend our earnest sympathy to those who were bound to him by the most endearing ties, and who, by this sad dispensation of an All-wise Providence, have been overwhelmed by the deepest affliction.

*Resolved*, That, in token of our sorrow for the loss of our associate, we attend his funeral in a body; and that the usual badge of mourning be worn by the members.

*Resolved*, That a copy of these Resolutions be sent to the family of Dr. Edmeston; and that they be published in the newspapers of this city; and that they be entered upon the records of the Society.

The Resolutions, being duly seconded, were voted upon, and were unanimously adopted.

Dr. C. D. MOSHER said: *Mr. President*,—I desire, with other members who have spoken, to express the profound respect I entertain for Dr. Edmeston. Only of late have I known him well: he was one who improved on acquaintance; he was a modest and unassuming physician. I desire to express my appreciation of his good qualities as a man and as a physician. In the early part of the rebellion young and inexperienced physicians went out as medical officers, and after the war, by the exercise of peculiar qualities not necessarily connected with their professional abilities, became very prominent. Even a drug clerk, having spent a certain time in the army, became a physician, and asked the favor of the public because he had been in the military service. But Dr. Edmeston, a modest physician, entered the army as assistant, soon became chief surgeon, and came back a modest surgeon. I wish to attest my appreciation of his conduct as being worthy of being followed by others. He really sacrificed himself by his service in the field, as much so as if he had lost a limb.

Adjourned.

